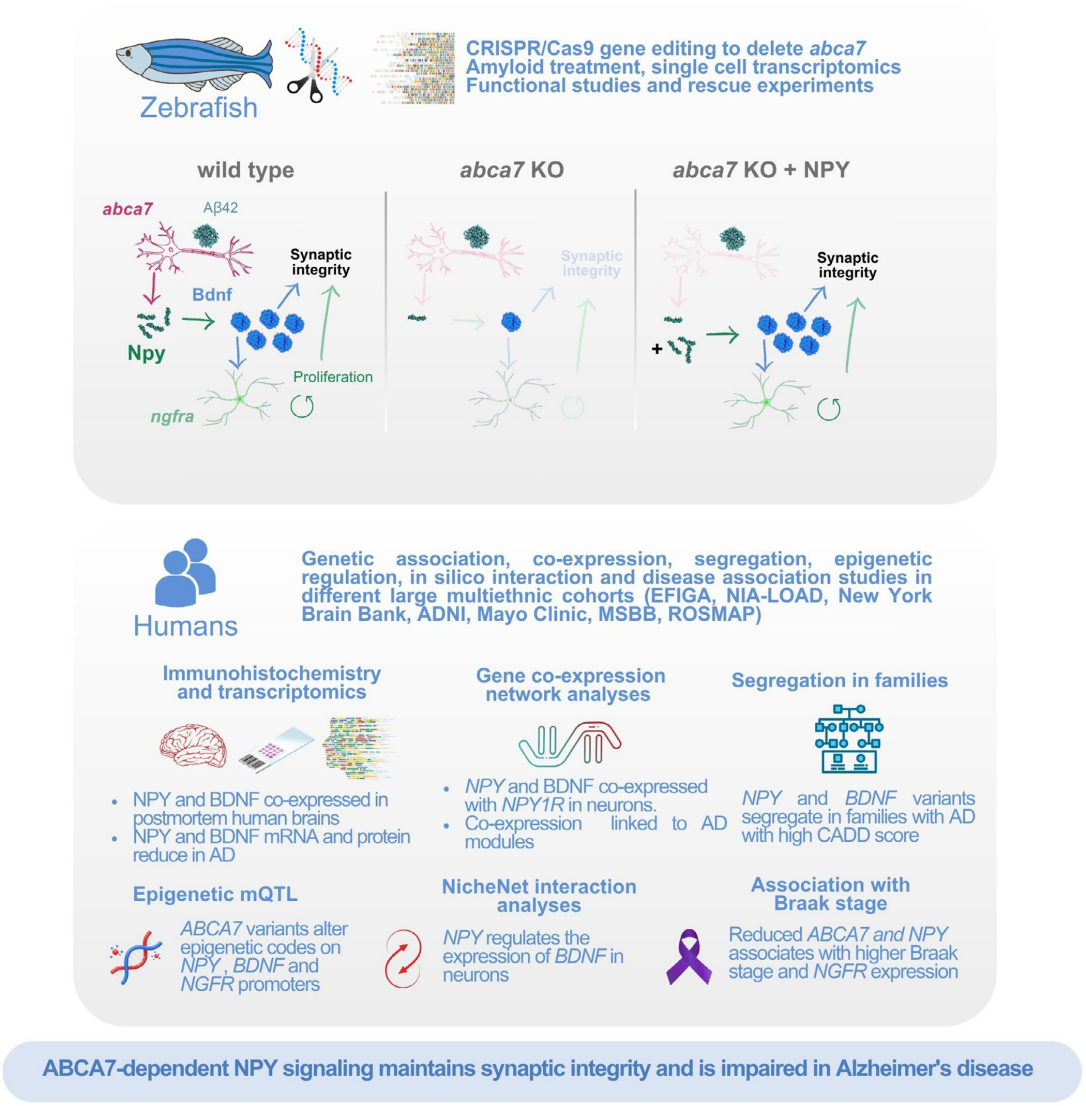# ABCA7‐dependent Neuropeptide‐Y signalling is a resilience mechanism required for synaptic integrity in Alzheimer’s disease

**DOI:** 10.1002/alz.089273

**Published:** 2025-01-03

**Authors:** Huseyin Tayran, Elanur Yilmaz, Prabesh Bhattarai, Yuhao Min, Xue Wang, Yiyi Ma, Nastasia Nelson, Nada Kassara, Mehmet I Cosacak, Ruya M Dogru, Dolly Reyes‐Dumeyer, Joseph S. Reddy, Min N Qiao, Delaney Flaherty, Zikun Yang, Tamil Iniyan Gunasekaran, Andrew F Teich, Badri N. Vardarajan, Giuseppe Tosto, Ozkan Is, Nilüfer Ertekin‐Taner, Richard Mayeux, Caghan Kizil

**Affiliations:** ^1^ Columbia University Irving Medical Center, New York City, NY USA; ^2^ Department of Neurology and The Taub Institute for Research on Alzheimer’s Disease and the Aging Brain, Columbia University Irving Medical Center, New York, NY USA; ^3^ The Taub Institute for Research on Alzheimer’s Disease and the Aging Brain, New York, NY USA; ^4^ Columbia University Irving Medical Center, New York, NY USA; ^5^ Mayo Clinic, Jacksonville, FL USA; ^6^ Taub Institute for Research on Alzheimer’s Disease and the Aging Brain, New York, NY USA; ^7^ Center for Translational & Computational Neuroimmunology, Department of Neurology, Columbia University Medical Center, New York City, NY USA; ^8^ German Center for Neurodegenerative Diseases, Dresden, SN Germany; ^9^ Gertrude H. Sergievsky Center, Vagelos College of Physicians & Surgeons, Columbia University, New York, NY USA; ^10^ Department of Neurology, Vagelos College of Physicians and Surgeons, Columbia University, New York, NY USA; ^11^ Taub Institute for Research on Alzheimer’s Disease and the Aging Brain, Columbia University, New York, NY USA; ^12^ Columbia University, New York, NY USA; ^13^ Taub Institute for Research on Alzheimer’s Disease and the Aging Brain, Vagelos College of Physicians and Surgeons, Columbia University, New York, NY USA; ^14^ The Gertrude H. Sergievsky Center, College of Physicians and Surgeons, Columbia University, New York, NY USA; ^15^ Department of Pathology and Cell Biology, New York, NY USA; ^16^ Department of Neurology, The New York Presbyterian Hospital, New York, NY USA; ^17^ The Taub Institute for Research on Alzheimer’s Disease and The Aging Brain, Columbia University, New York, NY USA; ^18^ Department of Neurology, Vagelos College of Physicians and Surgeons, Columbia University, and the New York Presbyterian Hospital, New York, NY USA; ^19^ G.H. Sergievsky Center, Vagelos College of Physicians and Surgeons, Columbia University, New York, NY USA; ^20^ Departments of Neurology, Psychiatry, and Epidemiology, Gertrude H. Sergievsky Center, The Taub Institute for Research on Alzheimer’s Disease and the Aging Brain, Vagelos College of Physicians and Surgeons, Columbia University, New York, NY USA; ^21^ Department of Neurology, Columbia University Medical Center, New York, NY USA; ^22^ The Taub Institute for Research on Alzheimer’s Disease and the Aging Brain, Vagelos College of Physicians & Surgeons, Columbia University, New York, NY USA; ^23^ Department of Neurology, College of Physicians and Surgeons, Columbia University, New York, NY USA

## Abstract

**Background:**

Genetic variations have emerged as crucial players in the etiology of Alzheimer’s disease (AD), and they serve for a better understanding of the disease mechanisms; yet the specific roles of these genetic variants remain uncertain. Animal models with reminiscent disease pathology could uncover previously uncharacterized roles of these genes. Therefore, we generated zebrafish models for AD variants to analyze the in depth molecular and biological functions of these variants.

**Method:**

Using CRISPR/Cas9, we generated a knockout model for *abca7*, orthologous to human *ABCA7*. We performed single cell transcriptomics and analyzed the altered genes and molecular pathways in zebrafish. We leveraged data from multiethnic AD cohorts at Mayo Clinic and Columbia University, to perform genetic association studies, co‐expression analyses, in silico interaction mapping, family based variant segregation analyses and epigenetic association studies, and the functional and histological studies in zebrafish.

**Result:**

The *abca7* ^±^ zebrafish reduced astroglial proliferation, synaptic integrity, and microglial response after Aβ42 toxicity. We found that the *abca7* loss‐of‐function (LOF) reduced neuropeptide Y (*npy*) expression as well as Brain‐derived neurotrophic factor (*bdnf)* and Nerve growth factor receptor *(ngfr)*. Human brain analysis showed reduced *NPY* in AD, regulatory interaction between *NPY* and *BDNF*, genetic variants in *NPY* associated with AD, and segregation of variants in *ABCA7*, *BDNF* and *NGFR* in families. *ABCA7* variants altered the epigenetic codes in *NPY*, *BDNF*, and *NGFR* promoter regions. Human results paralleled with zebrafish findings to indicate an evolutionarily conserved disease mechanism through ABCA7‐NPY signalling axis. NPY administration to zebrafish rescued the phenotypes in *abca7* knockout, suggesting a true biological relevance.

**Conclusion:**

Our results demonstrate a previously unknown link between *ABCA7* and *NPY* in regulation of synaptic integrity and neurogenesis in AD. We propose that ABCA7‐dependent NPY is a resilience factor in vertebrate brains, and this reserve mechanism is impaired in AD.